# Expression of Calcineurin Activity after Lung Transplantation: A 2-Year Follow-Up

**DOI:** 10.1371/journal.pone.0059634

**Published:** 2013-03-25

**Authors:** Sylvia Sanquer, Catherine Amrein, Dominique Grenet, Romain Guillemain, Bruno Philippe, Veronique Boussaud, Laurence Herry, Celine Lena, Alphonsine Diouf, Michelle Paunet, Eliane M. Billaud, Françoise Loriaux, Jean-Philippe Jais, Robert Barouki, Marc Stern

**Affiliations:** 1 Service de Biochimie Métabolomique et Protéomique, Hôpital Universitaire Necker-Enfants Malades Assistance Publique-Hôpitaux de Paris (AP-HP); INSERM UMR-S 747; and Université Paris Descartes, Centre Universitaire des Saints-Pères, Paris, France; 2 Service de Chirurgie Cardio-Vasculaire, Hôpital Européen Georges Pompidou, AP-HP, Paris, France; 3 Service de Pneumologie, Hôpital Foch, Suresnes, France; 4 Laboratoire de Biologie, Hôpital Foch, Suresnes, France; 5 Service de Pharmacologie, Hôpital Européen Georges Pompidou, AP-HP and Université Paris Descartes, Paris, France; 6 Service de Biochimie, Hôpital Européen Georges Pompidou, AP-HP, Paris, France; 7 Service de Biostatistiques, Hôpital Necker-Enfants Malades, AP-HP, Paris, France; Université Paris Descartes, Paris, France; University of California Los Angeles, United States of America

## Abstract

The objective of this pharmacodynamic study was to longitudinally assess the activity of calcineurin during the first 2 years after lung transplantation. From March 2004 to October 2008, 107 patients were prospectively enrolled and their follow-up was performed until 2009. Calcineurin activity was measured in peripheral blood mononuclear cells. We report that calcineurin activity was linked to both acute and chronic rejection. An optimal activity for calcineurin with two thresholds was defined, and we found that the risk of rejection was higher when the enzyme activity was above the upper threshold of 102 pmol/mg/min or below the lower threshold of 12 pmol/mg/min. In addition, we report that the occurrence of malignancies and viral infections was significantly higher in patients displaying very low levels of calcineurin activity. Taken together, these findings suggest that the measurement of calcineurin activity may provide useful information for the management of the prevention therapy of patients receiving lung transplantation.

## Introduction

Organ transplantation is the last alternative therapeutic option for selected patients with end-stage disease of a given organ. The survival of transplanted organs has markedly improved over the past few decades due to the use of immunosuppressive treatments. However, organ survival remains limited by the onset of chronic rejection and devastating adverse drug events. This is particularly true with lung transplantation. Despite increasing improvement in patient care, lung transplantation has the poorest outcomes mainly because of the development of chronic rejection in response to immunologic, ischemic and infectious injury [1]–[8]. Chronic rejection, which presents as a bronchiolitis obliterans syndrome (BOS), is defined as a progressive airflow obstruction and a deterioration of graft function. It accounts for more than 30% of all mortality after the third year following lung transplantation [8], [9]. Moreover, by promoting factor perivascular and peribronchial infiltration of activated lymphocytes into graft tissue, acute rejection remains an important risk factor for the development of BOS [10].

The standard for rejection prevention in lung transplantation consists of an immunosuppressive regimen which includes a calcineurin (CN) inhibitor (CNI) such as cyclosporine (CsA) and tacrolimus [11]. The CNI prophylactic dose is adjusted according to the whole blood concentration of the drug to avoid the occurrence of dose-dependent toxicities. However, the optimal balance of immunosuppression is difficult to achieve following transplantation. Inadequate immunosuppression may lead to transplant rejection and, on the other hand, excessive immunosuppression facilitates the development of severe complications such as infection or malignancy. To date, there are no robust biomarkers that allow the prediction of the extent of immunosuppression afforded by these treatments. This may be a partial explanation for the frequent failure of the immunosuppressive strategy after lung transplantation as illustrated by the facts that 50 to 60% of the patients develop acute rejection and up to 60% of the recipients who survive 5 years after transplantation are affected by BOS [8], [9], [12].

Different approaches have aimed at reducing the incidence and severity of acute rejection. As a first attempt, we have developed a pharmacodynamic approach for monitoring the extent of immunosuppression following transplantation. This approach is based on the activity of calcineurin, a calcium-calmodulin-dependent phosphatase. Calcineurin activity reflects the combination of the degree of T lymphocyte activation and the inhibitory effect of CNIs [13]–[17]. Calcineurin is a key factor involved during the early phase of T lymphocyte activation. When CN is activated, it dephosphorylates the nuclear factor of activated T cells (NFAT) which then allows translocation of NFAT into the nucleus. This leads to the synthesis of cytokines that are involved in T lymphocyte proliferation. It has been demonstrated clearly that T cell activation is dependent upon sustained calcium/CN signaling for maximal proliferation and cytokine production [18], [19]. Therefore, the CN activity (CN-a) measured in peripheral blood mononuclear cells (PBMCs) issued from allograft recipients receiving CNIs may be considered to be an index of T cell activation and a marker for graft-versus-host disease [20], [21].

In this study, we measured CN-a during the first 24 months after lung transplantation and we correlated the activities with the occurrence of acute rejection, BOS and adverse events which are known to be associated with over-immunosuppression, such as malignancies and infections.

## Materials and Methods

### Ethics Statement

The CALCILUNG study was a prospective observational study of lung transplant recipients. In accordance with French law, the study protocol was approved by the ethics committee of Paris-Broussais-HEGP. Patients enrolled in this study provided informed written consents.

### Patients

The study consisted of measuring CN-a during the first 24 months after lung transplantation. Patients were eligible if they were programmed to receive an immunosuppressive treatment consisting of the association of CsA, azathioprine and steroids. Patients followed a typical care regimen for post-lung transplantation patients, including surveillance fiberoptic bronchoscopy and bronchoalveolar lavage, spirometry, systematic transbronchial biopsies for acute rejection monitoring and blood sampling. The first surveillance biopsy was generally scheduled 7 days after transplantation. Subsequently, transbronchial biopsies were performed during the post-transplantation evaluation tests that were scheduled once a month up to the sixth month after lung transplantation and then every three months up to the 24^th^ month after transplantation. Spirometry, generally, was checked once a week up to the third month after lung transplantation, then once a month up to the first year after transplantation and then every three months. In general, the first CN-a assessment was performed before transplantation during the pre-transplantation evaluation tests. Post-transplantation CN-a measurements were performed at least once a month during the first 6 months after transplantation and then every three months. Sampling for CN-a measurements was concomitant to the other monthly scheduled post-transplantation evaluation tests. The transplantation characteristics of the patients enrolled in this study are listed in **Table 1**.

**Table 1:** Basal characteriwstics of patients.

	Total
	(n = 107)
**Age (yr)**	36±12
**Sex (M/F)**	64(60)/43(40)
**Initial disease**	
** cystic fibrosis**	64(60)
** emphysema**	19(18)
** others**	24(22)
** Type of transplantation (single/bilateral)**	16(15)/91(85)
**CMV mismatch at transplantaion (D+/R-)**	26(24)
**EBV mismatch at transplantation (D+/R-)**	7(7)
**Primary graft dysfunction grade III**	15±4
**Number of CN-a measurements/24 months**	6±3
**Time of follow-up (months)**	41±16
**Patients with acute rejection/6 months**	77(72)
**Patients with BOS grade ≥ I**	40(37)
**Patients with malignancies**	16(15)
**Patients with bacterial infections**	
** ≥1 episode**	63(59)
** ≥2 episodes**	31(29)
** ≥3 episodes**	15(14)
**Patients with viral infections**	
** ≥1 episode**	39(36)
** ≥2 episodes**	20(19)
** ≥3 episodes**	7(6.5)
**Patients with fungal infections**	
** ≥1 episode**	31(29)
** ≥2 episodes**	9(8.4)
** ≥3 episodes**	2(1.9)

Data are summarized as frequencies and percentage for categorical variables and as mean±SD for continuous variables. A total of 670 peripheral blood samples (mean of 6±3 samples per patient, range: 2–14) were obtained during the first 24 months following transplantation. Yr: year; M: male; F: female; EBV: empstein barr virus; CMV: cytomegalovirus; CN-a: calcineurin activity; BOS: bronchiolitis obliterans syndrome.

### Drug and Pharmacodynamic Monitoring

CsA and CN-a were both determined before the morning dose of CsA, when it was given orally. The clinical outcome of the patients was unknown to the biologist in charge of CN-a analyses and the results of the analyses were not given to the personnel (physicians and nurses) caring for the lung transplant patients. CsA was routinely measured with a locally available immunoassay. Mononuclear cells were isolated from the samples remaining by a Ficoll gradient method and CN-a measurements were made later. Briefly, 25 µg of proteins from mononuclear cells were incubated at 37°C for 30 min in the presence of phosphorylated RII peptide as a substrate of calcineurin. The dephosphorylation of the substrate was quantified by using high-performance liquid chromatography with ultraviolet detection as previously described [20]. Technical validation of this assay showed a correlation coefficient of the linear regression curves (linearity) greater than 0.9971 and a variation coefficient (inter-assay variability) less than 10% [20]. The stability of CN-a under our conditions was previously verified by performing pharmacokinetic and pharmacodynamic measurements over a 10-hr time course in stable renal transplant patients treated with CsA. A peak of inhibition of CN-a occurred at approximately the same time as the peak of CsA in whole blood, and the concentrations of both CN-a and CsA gradually returned to baseline levels [20].

### Diagnosis of Acute Rejection

Episodes of acute rejection were diagnosed on the basis of pulmonary function tests and histological evaluation of transbronchial biopsies. Acute rejection was graded according to the ISHLT criteria [22]. During the first six months following transplantation, treatment of acute rejection with steroids was initiated in patients with either an alteration or a 3-month stagnation of their pulmonary function and/or in patients for whom a grade A1 or higher was assessed based on their transbronchial biopies. Very few acute rejections of grade higher than A1 were diagnosed in these patients.

### Determination of Pulmonary Function

Pulmonary function was estimated from the spirometric data FEV1, representing the forced expiratory volume in one second. To assess the variation of pulmonary function versus time during the first six months after transplantation, FEV1 ratios were calculated from the ratio of the difference between two spirometric values obtained approximately 1 month apart to the number of days between two spirometric measurements. We expressed these ratios in liters per second per day. Because we considered positive FEV1 ratios as a normal evolution of pulmonary function, only null and negative values of FEV1 ratios, which reveal a negative alteration of pulmonary function were taken into account for the study.

### Diagnosis of Chronic Rejection/BOS

BOS was diagnosed and graded according to the ISHLT criteria [23]. BOS was defined as a sustained decrease of at least 20 percent in the FEV1 spirometric data as compared to the patient’s maximum values in the absence of other causes [23]. Azithromycin therapy was started in patients displaying a strong reduction in FEF_25–75_. In this study, we took into account the occurrence of BOS of grade I or higher.

### Statistical Analysis

The values are expressed as the means±SD or the medians and percentiles. For the evaluation of the relationship between CN-a and acute rejection, CN-a values were censored when patients received a first IV bolus of steroids. Kernel smoothing curves were generated and the dispersion of extreme CN-a values was determined. For the evaluation of the relationship between CN-a and pulmonary function, we compared the rates of negative altered FEV1 at different CN-a levels. The survival without BOS, overall survival and the occurrence of adverse events were estimated by the Kaplan-Meier method. Other potentially associated risk factors of BOS occurrence were evaluated by using a stepwise logistic regression model.

Analyses were performed by using SAS 9.2 and Graphpad Prism softwares. Two-tailed P<0.05 were deemed significant. In case of multiple group comparisons, p-values were adjusted by the Bonferroni method.

## Results

**Figure 1:**
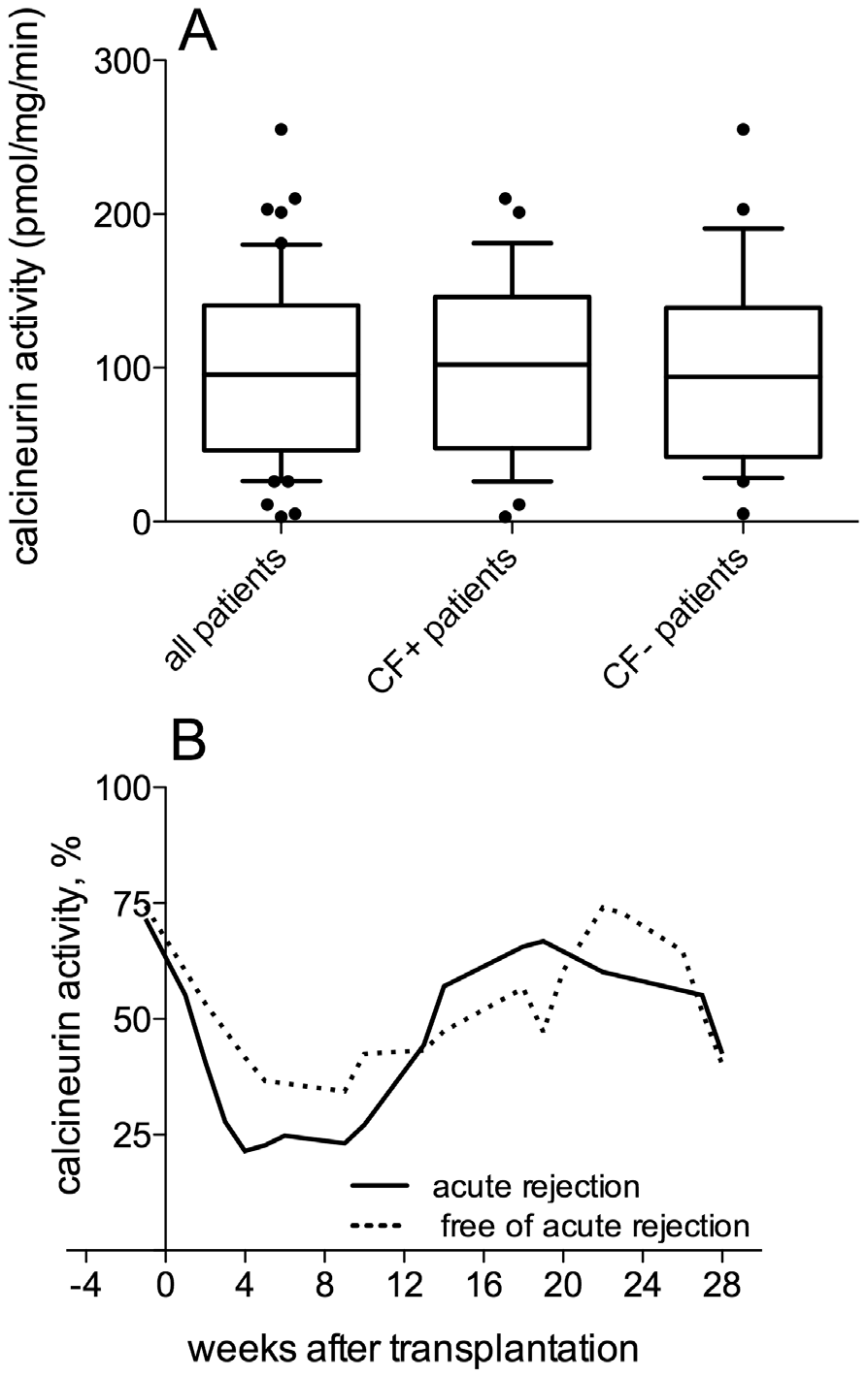
Calcineurin activity and acute rejection. (A) Calcineurin activity (CN-a) was measured before lung transplantation in 52 of the 107 patients enrolled in the participating center. The results are presented as box plots and 10–90 percentile whiskers. We compared CN-a expression prior to transplantation in patients with or without cystic fibrosis (CF) since it is the main initial end-stage lung disease that led to lung transplantation in this cohort of patients and a similar dispersion of the CN-a values was found in CF+ and CF- patients (p = 0.77, Mann-Whitney test). Subsequently, a relationship between extreme values of calcineurin activity and acute rejection was investigated. (B) Comparison across time of the median CN-a levels in patients displaying or not acute rejection: Kernel smoothing curves were generated. The 2 groups of patients displayed similar profiles of CN-a which consist of a phase of enzyme inhibition within the first 10 weeks after transplantation followed by a phase in which enzyme activity is restored. The phase of CN-a inhibition tended to be faster and more marked in patients who had developed acute rejection as compared to patients who were free of acute rejection. Similarly, the increase of enzyme activity to baseline levels tended to be faster and more pronounced in patients who had developed acute rejection.

From March 2004 to October 2008, 107 patients who received lung transplants were examined for CN-a monitoring. The initial clinical-biological characteristics of these patients are shown in **Table 1**. Patients were followed until 2009. A total of 670 blood samples (mean of 6±3 samples per patient, range: 2–14) were obtained during the first 24 months following transplantation. We compared the levels of CN-a prior to transplantation in patients with or without cystic fibrosis (CF) since this was the main initial end-stage lung disease that led to lung transplantation in this cohort of patients. There was no difference in the average pre-transplantation CN-a between patients with cystic fibrosis and the other patients (**Fig. 1A**).

### Calcineurin Activity and Acute Rejection

The relationship between CN-a and acute rejection was assessed during the first six months after transplantation since acute rejection mainly occurred during that period of time. Of the 107 lung-transplant recipients, 30 (28%) were free of any episode of acute rejection during the first 6 months after transplantation whereas 75 patients (71%) received a first rescue therapy by intravenous bolus (IV) of steroids following a diagnosis of acute rejection based either on their transbronchial biopsies for 68 of them (64%) or on their pulmonary function for 7 of them (7%). Furthermore, 2 patients (2%) were not rescued although an episode of acute rejection was diagnosed. The first episode of acute rejection occurred at a median time of 8 days (extreme values, 5–192 days). A large dispersion of CN-a values was observed, and since no reproducible pattern in CN-a could be discerned, we chose to express the CN-a data using their quartile range. For the patients enrolled in the study, the median of CN-a was 31 pmol/mg/min with values of 9, 12, 14, 17, 62, 84, 102 and 121 pmol/mg/min for the 10^th^, 15^th^, 20^th^, 25^th^, 75^th^, 80^th^, 85^th^ and 90^th^ percentiles, respectively (**Table 2**). We compared the median CN-a levels for the 2 groups of patients (with or without acute rejection) across time, and we generated Kernel smoothing curves (**Fig. 1B**). Although the 2 groups of patients displayed similar profiles of CN-a consisting of a phase of enzyme inhibition within the first 10 weeks after transplantation followed by a phase of restoration of enzyme activity, it appeared that the phase of CN-a inhibition tended to be faster and more marked in patients who developed acute rejection as compared to patients who did not develop acute rejection. Similarly, the increase of enzyme activity, almost to baseline levels, tended to be faster and more pronounced in patients who had developed acute rejection (**Fig. 1B**). We examined the dispersion of CN-a values in the 2 groups of patients by counting the number of values below the 10^th^ to the 25^th^ percentiles and above the 75^th^ to the 90^th^ percentiles (**Table 2**). We found that the number of CN-a values below the 10^th^–25^th^ percentiles was much higher in the group of patients who had developed acute rejection during the first 10 weeks after transplantation as compared to those that did not develop acute rejection [for example, 7 patients (15%) and 19 patients (40%) with acute rejection vs 2 patients (6%) and 6 patients (17%) free of acute rejection below the 10^th^ and 25^th^ percentiles respectively, **Table 2**]. Similarly, the number of CN-a values above the 75^th^–90^th^ percentiles was higher in the group of patients who had developed acute rejection during the phase of enzyme activity restoration between 13 to 28 weeks after transplantation [9 patients (35%) and 5 patients (19%) with acute rejection vs 19 patients (30%) and 7 patients (10%) free of acute rejection above the 75^th^ and 90^th^ percentiles respectively, **Table 2**].

**Table 2:** Dispersion of the values of calcineurin activity.

	1 to 10 weeks after transplantation	13 to 28 weeks after transplantation
**Patients free of acute rejection**		
**number of CN-a determinations**	35	68
**number of CN-a :**		
**- ≤10^th^ percentile** (9 pmol/mg/min)	2(6)	7(10)
**- ≤15^th^ percentile** (12 pmol/mg/min)	2(6)	11(16)
**- ≤20^th^ percentile** (14 pmol/mg/min)	3(9)	14(21)
**- ≤25^th^ percentile** (17 pmol/mg/min)	6(17)	18(26)
**- ≥75^th^ percentile** (62 pmol/mg/min)	6(17)	19(30)
**- ≥80^th^ percentile** (84 pmol/mg/min)	5(14)	14(21)
**- ≥85^th^ percentile** (102 pmol/mg/min)	4(11)	11(16)
**- ≥90^th^ percentile** (121 pmol/mg/min)	2(6)	7(10)
**Patients with acute rejection**		
**number of CN-a determinations**	48	26
**number of CN-a :**		
**- ≤10^th^ percentile** (9 pmol/mg/min)	7(15)	1(4)
**- ≤15^th^ percentile** (12 pmol/mg/min)	10(21)	2(8)
**- ≤20^th^ percentile** (14 pmol/mg/min)	14(29)	2(8)
**- ≤25^th^ percentile** (17 pmol/mg/min)	19(40)	2(8)
**- ≥75^th^ percentile** (62 pmol/mg/min)	5(10)	9(35)
**- ≥80^th^ percentile** (84 pmol/mg/min)	4(8)	8(31)
**- ≥85^th^ percentile** (102 pmol/mg/min)	4(8)	5(19)
**- ≥90^th^ percentile** (121 pmol/mg/min)	2(4)	5(19)

Data are summarized as frequencies and percentage. A total of 103 measurements of calcineurin activity (CN-a) were performed in the group of patients free of acute rejection and of 74 in the group of patients with acute rejection before the occurrence of this event.

### Calcineurin Activity and Pulmonary Function

We next examined whether extreme CN-a values were associated with an alteration in pulmonary function during the first 6 months following transplantation. A higher per cent of altered FEV1 ratios was found in patients displaying CN-a values out of the range of 17–62 pmol/mg/min corresponding to the 25^th^ and 75^th^ percentiles as compared to patients displaying values of CN-a within this range [42 (48%) vs 27 (34%), respectively, **Table 3**]. A similar finding was observed for patients having CN-a values out of the range of 12–102 pmol/mg/min corresponding to the 15^th^ and 85^th^ percentiles as compared to patients displaying CN-a values within this range [26 (49%) vs 43 (38%), respectively, **Table 3**]. On the basis of these results, we chose to compare the long-term outcomes of patients who displayed CN-a values within the range of 12–102 pmol/mg/min versus patients who exhibited at least one CN-a value outside this range during the first 24 months after transplantation.

**Table 3:** Calcineurin activity and pulmonary function.

range of CN-a levels	number of determinations	number of altered FEV1-ratio
**25th - 75th percentile** (17–62 pmol/mg/min)		
** 25th–75th in**	79	27(34)
** 25th–75th out**	87	42(48)
**20th–80th percentile** (14–84 pmol/mg/min)		
** 20th–80th in**	96	36(38)
** 20th–80th out**	70	33(47)
**15th–85th percentile** (12–102 pmol/mg/min)		
** 15th–85th in**	113	43(38)
** 15th–85th out**	53	26(49)
**10th–90th percentile** (9–121 pmol/mg/min)		
** 10th–90th in**	125	52(42)
** 10th–90 out**	41	17(41)

Data are summarized as frequencies and percentage. The relationship between calcineurin Activity (CN-a) and the forced expiratory volume in one second (FEV1) ratio was studied from A total of 166 values collected from 87 patients (mean of 2±1 data per patient, range : 1–5).

### Adverse Events Related to Over-immunosuppression

**Figure 2:**
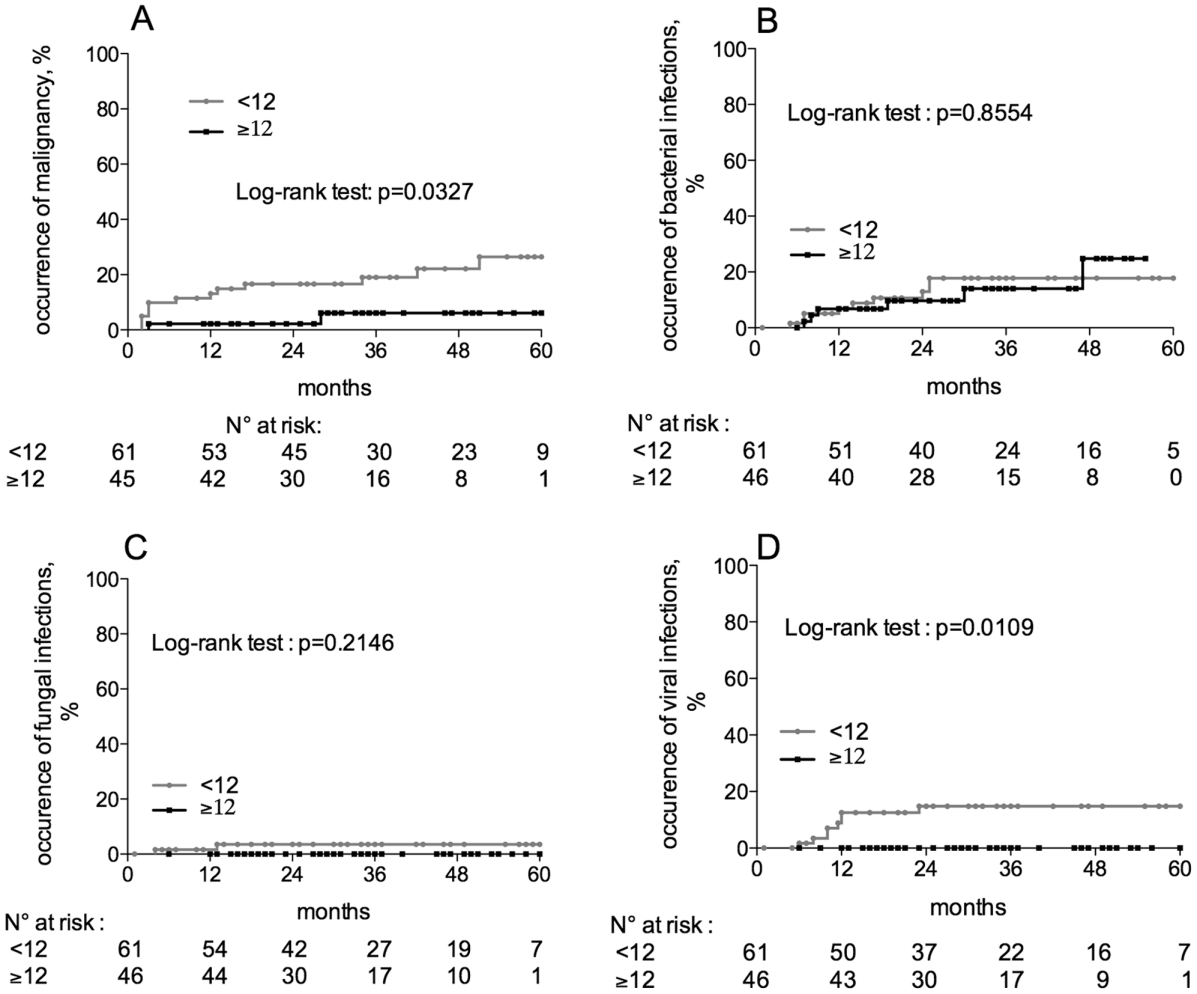
Calcineurin activity and adverse events related to over-immunosuppression. The onset of events known to be related to over-immunosuppression, such as malignancies and infections, was compared between patients displaying or not low CN-a levels by the Kaplan and Meier method. (A) CN-a and malignancies: the occurrence of malignancies was significantly higher in patients displaying at least one CN-a value below 12 pmol/mg/min during the first 24 months after transplantation as compared to patients with higher CN-a values (28% vs 6%, p = 0.0218, Log-rank test). The examination of the relationship between CN-a and infections was performed by separating the infections of bacterial, viral and fungal origin. (B) CN-a and bacterial infections: the occurrence of 3 episodes of bacterial infections was similar in the 2 groups of patients (18% vs 25%, p = 0.85, Log-rank test). (C) CN-a and fungal infections: the occurrence of 3 episodes of fungal infections was similar in the 2 groups of patients (3.5% vs 0%, p = 0.21, Log-rank test). (D) CN-a and viral infections: the occurrence of 3 episodes of viral infections was significantly higher in patients displaying at least one CN-a value below 12 pmol/mg/min during the first 24 months after transplantation compared to patients with higher CN-a values (15% vs 0%, p = 0.01, Log-rank test).

Because low CN-a levels might reflect an over-immunosuppression, we compared the onset of events known to be associated with over-immunosuppression, such as malignancies and infections, between patients displaying or not CN-a levels below the lower threshold of 12 pmol/mg/min during the first 24 months after transplantation. Of the 107 lung-transplant recipients in this study, 1 patient, who displayed an Epstein-Barr virus-induced lymphoma before any CN-a measurement was made, was not considered for the evaluation of the relationship between CN-a and malignancies. The occurrence of malignancies was significantly higher in patients displaying at least one CN-a value below 12 pmol/mg/min as compared to patients with higher CN-a values (28% vs 6%, p = 0.0218, Log-rank test, **Fig. 2A**). The assessment of the relationship between CN-a and infections was performed on the 107 patients enrolled in the study by separating the infections of bacterial, viral and fungal origin. The occurrence of bacterial and fungal infections was similar in the 2 groups of patients (**Fig. 2B,C**) whereas that of viral origin was significantly higher in patients displaying at least one CN-a value below 12 pmol/mg/min as compared to patients with higher CN-a levels (15% vs 0%, p = 0.0109, Log-rank test, **Fig. 2D**). This finding was restricted to patients with at least 3 episodes of viral infection (**Fig. 2D**).

### Calcineurin Activity and BOS/chronic Rejection

**Figure 3:**
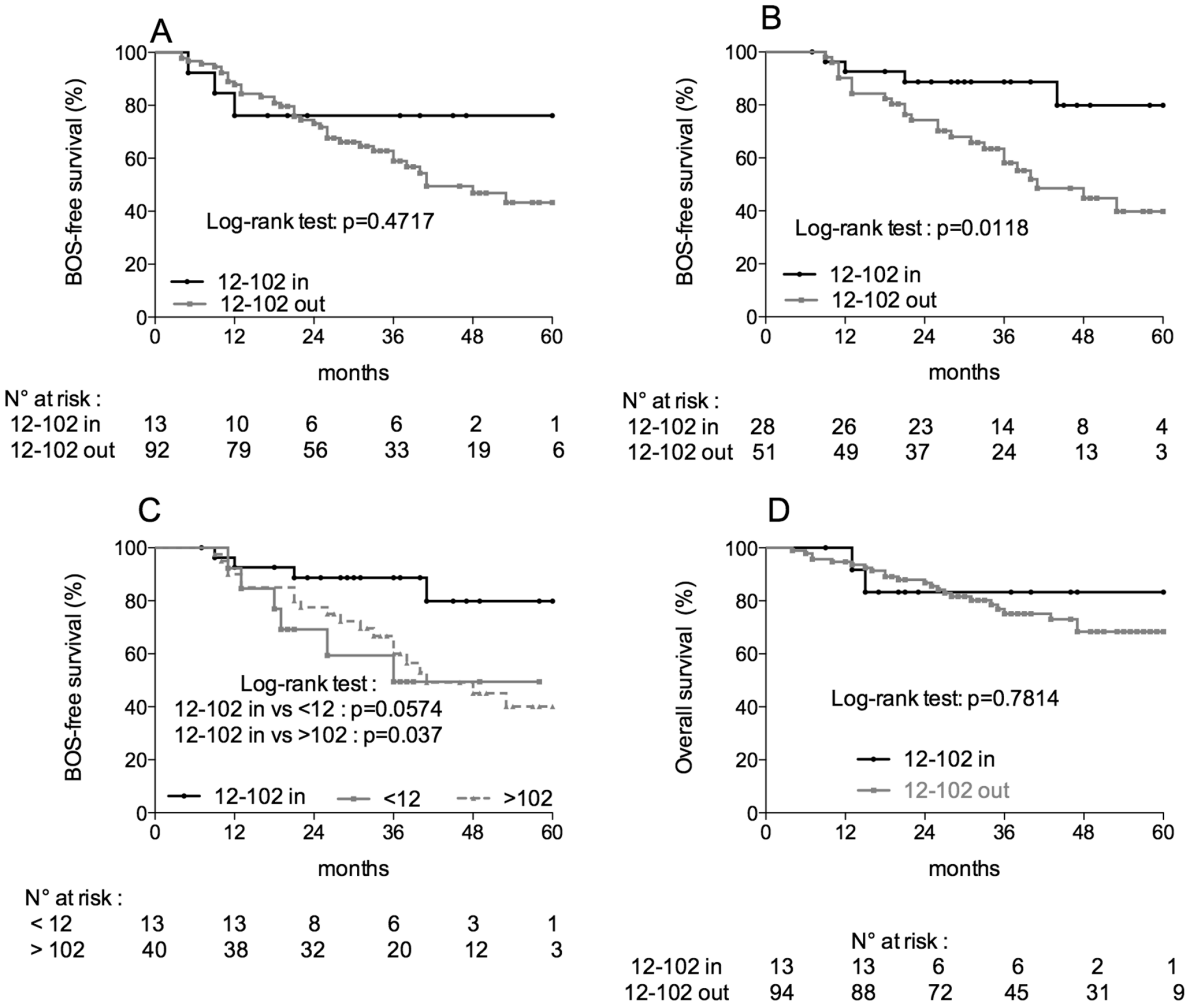
Calcineurin activity, BOS and overall survival. BOS-free survival was estimated at 5 years after transplantation by the Kaplan and Meier method. (A) Calcineurin activity (CN-a) monitoring during the first 24 months after transplantation: although not statistically significant, the survival without BOS was higher in patients who displayed CN-a levels within the range of 12–102 pmol/mg/min as compared to patients who exhibited at least one CN-a value outside this range of 12–102 pmol/mg/min during the first 24 months following transplantation (76% vs 43%, p = 0.4717, Log-rank test). (B) CN-a monitoring from the 6^th^ month to the 24^th^ month after transplantation: the survival without BOS was significantly higher in patients who displayed CN-a levels within the range of 12–102 pmol/mg/min as compared to that of patients who exhibited at least one CN-a value outside this range from the 6^th^ month to the 24^th^ months following transplantation (80% vs 40%, p = 0.0118, Log-rank test). (C) CN-a monitoring from the 6^th^ to the 24^th^ month after transplantation: the threshold values were further separated in 2 groups : <12 pmol/mg/min, >102 pmol/mg/min. The BOS-free survival in patients from each of these groups was compared to that from patients who displayed CN-a levels within the range of 12–102 pmol/mg/min. A significant reduction of the survival without BOS was found in patients who displayed CN-a levels higher than 102 pmol/mg/min (40% vs 80%, p = 0.037, Log-rank test), whereas a reduction in BOS-free survival in the limit of statistical significance was found in patients who displayed CN-a levels lower than 12 pmol/mg/min (49% vs 80%, p = 0.0574, Log-rank test). (D) Calcineurin activity and overall survival: no significant difference was found in the overall survival between the 2 groups of patients exhibiting calcineurin activity levels within or outside of the range of 12–102 pmol/mg/min.

CN-a was monitored the first 24 months after transplantation. We tested for the presence of a relationship between CN-a and the occurrence of BOS. Of the 107 lung-transplant recipients that were studied, 2 patients, who displayed a bronchopulmonary carcinoma and for whom pulmonary function tests were not performed, were not included in this analysis. The median time of follow-up for the patients was of 32.3 months with extreme values of 4–60 months, and that of the occurrence of BOS was of 19 months with a range of 4–53 months. BOS was diagnosed in 14 patients (13%), 35 patients (33%) and 41 patients (38%) 12 months, 36 months and 60 months after transplantation, respectively. Although not statistically significant, the survival without BOS was longer in patients who displayed CN-a levels within the range of 12–102 pmol/mg/min as compared to patients who exhibited at least one CN-a value outside this range (76% vs 43%, p = 0.4717, Log-rank test, **Fig. 3A**). Interestingly, very few patients displayed CN-a values within the range of 12–102 pmol/mg/min throughout the 24-month period of monitoring of CN-a [13 patients (12%) with CN-a values within the range vs 92 patients (88%) with at least one CN-a value out of the range, **Fig. 3A**]. This distribution of values between the two groups of patients (within vs outside of the range 12–102 pmol/mg/min) made it very difficult to determine whether a statistically significant difference in BOS-free survival exists for the groups.

In addition and because BOS was diagnosed mostly after sixth months after transplantation, we restricted the analysis of a relationship between CN-a and BOS to CN-a data obtained between 6 and 24 months after transplantation. Due to the absence of data or the occurrence of BOS before the 6^th^ month after transplantation, 26 patients were not considered in this analysis. With these restrictions, the patients were distributed more evenly between the 2 groups [28 patients (35%) with CN-a values within the range of 12–102 pmol/mg/min vs 51 patients (65%) with at least one CN-a value outside of the range, **Fig. 3B**]. BOS-free survival was found to be significantly higher in patients who displayed CN-a levels within the range of 12–102 pmol/mg/min as compared to patients who exhibited at least one CN-a value outside this range from the 6^th^ month to the 24^th^ month following transplantation (80% vs 40%, p = 0.0118, Log-rank test, **Fig. 3B**). In addition, we have determined whether known risk factors of BOS were involved in the association of CN-a values with BOS during this 18-month period of CN-a monitoring. The association between BOS and CN-a was not significantly accounted for by the following potential risk factors: acute rejection, CMV infection, primary graft dysfunction grade III, anti-HLA antibodies, gastro-oesophageal reflux. In a logistic regression model taking into account the other risk factors, the CN-a range was the only variable significantly associated with BOS (odds ratio 5.7, 95% CI [1.7–19.2], p = 0.045).

Since CN-a levels lower than 12 and higher than 102 pmol/mg/min suggested two putatively different mechanisms by which BOS developed, we next separated these threshold values to determine whether the groups had similar, significant decreases in survival without BOS. A significant reduction in BOS-free survival was found in patients who displayed CN-a levels higher than 102 pmol/mg/min (40% vs 80%, p = 0.037, Log-rank test, **Fig. 3C**), whereas a reduction in BOS-free survival in the limit of statistical significance was found in patients who displayed CN-a levels lower than 12 pmol/mg/min (49% vs 80%, p = 0.0574, Log-rank test, **Fig. 3C**).

### Calcineurin Activity and Overall Survival

Of the 107 patients enrolled in the study, 25 patients (23%) died during follow-up. At this time of the evaluation, no significant difference was found in the overall survival between the 2 groups of patients exhibiting CN-a levels within or outside of the range of 12–102 pmol/mg/min (**Fig. 3D**). This relationship should be re-assessed after a longer period of follow-up.

### Calcineurin Activity and Cyclosporine Blood Levels

**Figure 4:**
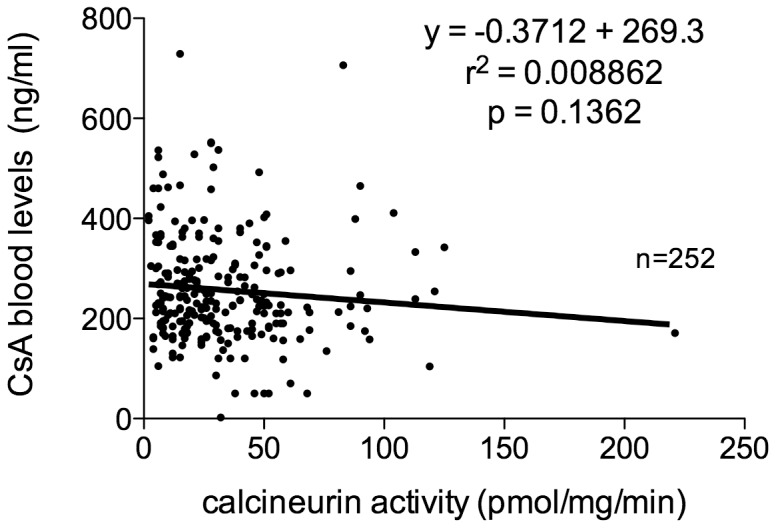
Calcineurin activity and cyclosporine blood levels. The relationship between calcineurin activity (CN-a) and the levels of cyclosporine (CsA) in blood was investigated. No correlation was found between CN-a and the level of CsA in blood.

We next investigated whether CsA blood levels could explain the modification in CN-a that we have observed. However, as shown in **Figure 4**, we did not find any significant correlation between CN-a and CsA blood levels, as also observed in other types of transplantation [20], [21], [24].

## Discussion

The activity of calcineurin measured in the PBMCs of allograft recipients who received inhibitors of calcineurin has been shown to be an index of T cell activation and a marker for graft-versus-host disease [20], [21]. It was thought that a high CN-a reflected poor immunosuppression whereas a low CN-a reflected potent immunosuppression. Therefore, our working hypothesis, for the present study, was that the level of CN-a can predict the degree of immunosuppression after lung transplantation, and, thus, be useful for predicting both the occurrence of rejection, related to an inadequate immunosuppression, and the development of severe complications, related to excessively potent immunosuppression. However, we report here that patients who displayed extreme CN-a values, either high or low values, were mainly those patients who developed acute rejection and had an altered pulmonary function. These observations led us to define an optimal activity for CN between two thresholds, 12 and 102 pmol/mg/min. Patients who had CN-a values within this range had a significantly higher survival without BOS. Furthermore, the occurrence of malignancies and viral infections was significantly lower in patients who exhibited CN-a values higher than 12 pmol/mg/min.

With the introduction of more potent immunosuppressive agents and newer combinations during the last ten years, patient and graft survivals have dramatically increased following most types of solid organ transplantation. However the incidence of post-transplantation infections and cancer also has increased. It was thought that potent immunosuppression, as reflected by the occurrence of adverse events, was protective against immunogenic stimulation. However, despite a modern immunosuppressive regimen, lung transplantation is characterized by both poor patient and graft survivals as well as devastating adverse events. The results of the present study may provide a partial explanation for the disappointing long-term outcomes in lung transplant patients. Indeed, we observed extreme CN-a values, below 12 pmol/mg/min or higher than 102 pmol/mg/min, more frequently in the present cohort of lung transplant patients than in other types of transplant patients that we have examined such as hematopoietic stem cell transplant patients [20] or heart transplant patients (unpublished data). In addition, we established a relationship between CN-a and the occurrence of both acute and chronic rejection. This relationship was non-monotonic in that both very low and very high CN-a levels were associated with the onset of acute rejection. As expected, very high CN-a could reflect poor immunosuppression that is not sufficient to counteract the immunogenic activation of T lymphocytes.

On the contrary, the presence of a low threshold was very surprising since very low CN-a levels should have been associated with a strong protection against lymphocyte activation. In fact, the patients with very low CN-a levels were, indeed, strongly immunosuppressed since they developed a higher rate of both malignant diseases and viral infections as compared to patients with higher CN-a levels. Nevertheless, their CN-a levels did not reflect their immunologic potency towards the graft. This finding is consistent with the recently reported activation of a negative feedback loop, via endogeneous CN inhibitors, calcipressins, which down-regulate the CN/NFAT signaling pathway when it is activated [25]–[28]. Although the calcipressin family has been extensively investigated in brain, heart and endothelial cells, a very limited number of studies has been reported concerning the immune system. Additionally, the impact of calcipressins on the effects of immunosuppressive agents in the context of transplantation has never been assessed. Therefore, we anticipate that low CN-a levels displayed by lung transplant patients developing a rejection are associated first with a lymphocyte activation subsequently followed by a strong endogenous down-regulation of the calcineurin/NFAT signaling pathway.

Taken together, these findings on the relationships between CN-a and acute rejection, pulmonary function and the occurrence of adverse events related to over-immunosuppression led us to define an optimal activity for CN between two thresholds, 12 and 102 pmol/mg/min, and to assess, retrospectively, whether patients who displayed CN-a values between these two thresholds had a significantly higher rate of survival without BOS/chronic rejection. Lung transplantation is the type of organ transplantation that gives the poorest outcomes, with 45% of the recipients dying within 5 years, mainly due to the development of chronic rejection in response to immunologic, ischemic and infectious injury. Unfortunately, once the clinical signs of BOS/chronic rejection appear, it is usually too late to reverse it. We report here that patients who displayed CN-a values within the range of 12–102 pmol/mg/min had a significantly higher rate of survival without BOS/chronic rejection. In addition, this association of BOS/chronic rejection and CN-a values was not explained by the known risk factors of BOS such as acute rejection, CMV infection, primary graft dysfunction grade III, anti-HLA antibodies or gastro-oesophageal reflux. In a logistic regression model taking into account the other risk factors, the CN-a range was the only variable significantly associated with BOS/chronic rejection. Therefore, CN-a may constitute an additional risk factor of BOS. Currently, overall survival is not significantly associated with CN-a values. However, we have to take into account that, in our study, the median time of occurrence of BOS was 19 months and that it has been shown that the median survival after the onset of BOS is 30 months [29]. Therefore, the overall survival according to CN-a values need to be re-assessed after a longer period of follow-up.

The degree of CN inhibition up to 12 hours after treatment with CsA has been shown to vary directly with the blood levels of CsA [30]. However, this relationship might not persist after several months of treatment with CsA because of the potential contribution of lymphocyte stimulation to the drug effect upon the target. Indeed, we report here that blood levels of CsA and trough levels of CN-a are not correlated. This observation is in agreement with previous findings in hematopoietic stem-cell transplant-, in liver transplant- and in kidney transplant-patients [20], [21], [24], [31], [32]. However, the absence of correlation between CN-a levels and CsA whole blood concentrations does not mean that the latter is not a predictor of patient outcome.

There are advantages and limitations to using CN-a as a biomarker. First, interpretation by clinicians of CN-a values, which appear to display a considerable dispersion, may prove to be difficult. Second, other markers, such as the degree of T-cell activation in blood [33], in broncho-alveolar fluid [34], [35] or increased T-cell pro-inflammatory cytokine production in the graft [36], have been associated with acute rejection. However, as compared to these studies, the main objective of our study was to identify and characterize a rejection marker that is the most directly related to the degree of immunosuppression produced by an anticalcineurin drug such as CsA. Indeed, only this type of marker aids in determining therapeutic options. Consequently, we believe that our observations can help clinicians in their use of CsA. Our findings suggest that CsA should be administered with much more caution during episodes of acute rejection than might have been thought previously. In particular, monitoring of CN-a sequentially after transplantation might be helpful for facilitating the optimization of multidrug immunosuppressant regimens including those employing CsA. The targeting of CN-a levels between the 25^th^ and 75^th^ percentiles, that is between 17 and 62 pmol/mg/min, can be proposed as a desirable therapeutic range in order to avoid values of CN-a outside the range of 12–102 pmol/mg/min that is associated with poor outcome. Indeed, the dose of CsA should be increased for patients with suspected acute rejection and CN-a levels over 62 pmol/mg/min but not for those patients with CN-a levels below 17 pmol/mg/min. In the latter case, a switch to another class of immunosuppressant can be recommended. Recommendations of this type can be made only when the most specific biomarkers are used and not when general biomarkers, only, are available. However, our data are still preliminary and need to be confirmed through a prospective validation cohort. Further investigation of calcineurin levels need to be carried before considering calcineurin levels as a biomarker.

In summary, we have shown that a relationship exists between CN-a and both acute and chronic rejection in lung transplant patients. Further, we have defined an optimal activity for calcineurin between two thresholds : the risk of rejection was higher when the enzyme activity was above the upper threshold of 102 pmol/mg/min or below the lower threshold of 12 pmol/mg/min. In addition, we report that the occurrence of malignancies and viral infections was significantly higher in patients displaying CN-a below the lower threshold. Based upon these findings, CN-a appears as a potential predictive biomarker that could lead to new guidelines for the management of lung transplant patients.
